# Object-based feedback attention in convolutional neural networks improves tumour detection in digital pathology

**DOI:** 10.1038/s41598-024-80717-3

**Published:** 2024-12-05

**Authors:** Andrew Broad, Alexander Wright, Clare McGenity, Darren Treanor, Marc de Kamps

**Affiliations:** 1 School of Computing, University of Leeds, Leeds, UK; 2 Leeds Institute for Data Analytics, University of Leeds, Leeds, UK; 3 Leeds Teaching Hospitals NHS Trust, Leeds, UK; 4 Division of Pathology and Data Analytics, Leeds Institute of Medical Research, University of Leeds, Leeds, UK; 5 National Pathology Imaging Cooperative, Leeds, UK; 6 University of Leeds, Leeds, UK; 7 Department of Clinical Pathology, Linköping University, Linköping, Sweden; 8 Department of Clinical and Experimental Medicine, Linköping University, Linköping, Sweden; 9 Center for Medical Image Science and Visualization (CMIV), Linköping University, Linköping, Sweden

**Keywords:** Medical research, Computer science, Software

## Abstract

Human visual attention allows prior knowledge or expectations to influence visual processing, allocating limited computational resources to only that part of the image that are likely to behaviourally important. Here, we present an image recognition system based on biological vision that guides attention to more informative locations within a larger parent image, using a sequence of saccade-like motions. We demonstrate that at the end of the saccade sequence the system has an improved classification ability compared to the convolutional neural network (CNN) that represents the feedforward part of the model. Feedback activations highlight salient image features supporting the explainability of the classification. Our attention model deviates substantially from more common feedforward attention mechanisms, which linearly reweight part of the input. This model uses several passes of feedforward and backward activation, which interact non-linearly. We apply our feedback architecture to histopathology patch images, demonstrating a 3.5% improvement in accuracy (*p* < 0.001) when retrospectively processing 59,057 9-class patches from 689 colorectal cancer WSIs. In the saccade implementation, overall agreement between expert-labelled patches and model prediction reached 93.23% for tumour tissue, surpassing inter-pathologist agreement. Our method is adaptable to other areas of science which rely on the analysis of extremely large-scale images.

## Introduction

Digital pathology (DP) systems enable the rapid capture, sharing and viewing of Whole Slide Images (WSIs) at multi-gigapixel resolution, allowing detailed inspection of tissue samples for diseases such as cancer^1^. Diagnostic calculations based on pathology features such as Tumour/Stroma Ratio (TSR) can help to predict disease progression^2–4^ but require labour-intensive cell-counting over tens or hundreds of sampling points^5^. For such activities, pathologist-pathologist agreement rates are typically 85%^6^. A worldwide shortage of trained pathologists^6^ highlights the value of automated processing using Artificial Intelligence (AI).

AI image processing models such as Convolutional Neural Networks (CNNs) typically operate at a much smaller scale than the WSI^7,8^, often 224$\:\times\:$224 pixels. The WSI is typically 100,000$\:\times\:$80,000 pixels and is often processed by sampling multiple smaller patches, either by simply dividing the whole WSI into a grid of image tiles, or more economically by using iterative sampling^9,10^ or Sequential Patching^11^ methods. Diagnostic information about the WSI can be derived from a sufficient number of patch-wise outputs. For example, Multi-Instance Learning (MIL) systems^12,13^ categorise the WSI by grouping ‘bags’ of feature embeddings obtained by applying a CNN to each tile in the WSI.

Our proposed models operate at a patch scale. We introduce novel methods for patch-level feature extraction and classification, and for object (tumour) location in larger tiles of the WSI using a saccade-like process. These are proposed for use within established WSI-sampling pipelines.

Performance in patch processing is enhanced using attention. Attention allows humans and animals to focus on features of interest in a busy, high-resolution scene. *Bottom-up* attention^14^ uses biasing signals derived from lower layers in the visual stream, such that representations of objects of prior interest are passed preferentially to higher cognitive regions. This process is widely emulated in feedforward attention neural networks in DP^15^ and other imaging domains^16,17^, and in natural language processing^18^.

Contrastingly, our work explores the use of *top-down* attention^14,19^ to enhance performance in image analysis. In biological top-down attention, executive brain regions send signals back down the visual stream, selectively boosting or inhibiting responses to colours, textures and shapes associated with the target object.

This has previously been simulated in goal-directed^20^ and feedback-based neural networks^21–23^. Tsuda et al.^23^ demonstrate a U-Net segmentation model^24^ enhanced with top-to-bottom feedback-generated spatial attention in the input layer, which gave improved segmentation performance with colorectal cancer (CRC) pathology patches. Kubilius et al.^21,22^ published the CORnet CNN series for image classification, which used recurrent feedback loops within convolutional groups emulating V1, V2, V4 and Inferior-Temporal (IT) primate brain regions^25^. This feedback mechanism was shown to improve classification performance, particularly with deliberately cluttered, heterogeneous input images.

Existing attention models were reviewed for use at a patch scale, downstream from sampling from the WSI. Many models, including Transformers^18^, use Self-Attention (SA) modules which combine Query, Key and Value terms using scaled dot-product multiplication to generate the output attention vector. This requires $\:O\left({N}^{2}\right)$ neurons for pixel count N, or $\:O\left({W}^{4}\right)$ in terms of image width W. This becomes computationally expensive, even within common patch sizes, especially in the high-dimensional lower layers of a CNN incorporating SA. Tsuda et al.^23^ showed that replacing SA modules with multiplicative attention modules reduced model size, with only a marginal reduction in accuracy. Our FAL-CNN model uses this more efficient approach when applying feedback attention, thus regulating the total number of model weights.

Our novel Feedback Attention Ladder CNN (FAL-CNN) model combines multiple region-level feedback loops with top-to-bottom feedback generated using a U-Net decoder structure and applied at multiple levels in the feedforward CNN. These feedback paths are applied to a VGG19^8^-based feedforward CNN ‘backbone’ via multiplicative attention modules. VGG19 was chosen for its performance in pathology benchmarking tests^9^ and for its flexible sequential architecture which supported the incremental addition of feedback elements during development, although we will argue that our approach can be applied to other feedforward CNN architectures.

Our FAL-CNN exhibits recurrent behaviour, combining features from multiple iterations of the feedforward path, before and after feedback is applied, in a Feature Embedding Store (FES). This is analogous to the hidden vector in a recurrent neural network (RNN) such as the Long Short-Term Memory (LSTM)^26^. However, our FES stores the results from a finite number of feedforward and feedback cycles, rather than cyclically incorporating a hidden vector from each previous iteration, as would be done in a typical sequence-predicting RNN such as the LSTM.

We demonstrate that our feedback attention model delivers significant performance gains relative to the feedforward-only model across disparate data sets. We further show that the feedback activations highlight image regions that correspond to salient features in the input scene.

Attention in the animal kingdom also involves *saccade* behaviour, where executive brain regions direct a series of rapid eye movements to align the higher-resolution central fovea with features of interest in a larger scene. This approach uses a lower bandwidth than processing the whole input at full resolution^27^.

We emulated this process with a Saccade Model which resamples the input patch from a larger background region, which is available in the WSI, using attention distributions from FAL-CNN to align the centre of attention (CoA) at patch centre where our classifier is most sensitive. Expert re-labelling of the resampled patches was performed to assess the attention model’s updated predictions, confirming that the Saccade Model converges on regions of informative tissue such as tumour.

## Results

### Data extraction

We extracted 59,057 patches from 689 colorectal cancer WSIs originating from the QUASAR trial^28^ and follow-on studies^4,29^, at locations specified in ground truth labelling data from the latter work, into directories corresponding to the labelled class. Patches of $\:224\times\:224$px and $\:448\times\:448$px were extracted, respectively for model training and for evaluation of our saccade model. Patches were grouped by parent WSI and allocated to five test/training splits for five-fold cross-validation of subsequently trained models.

**Fig. 1:**
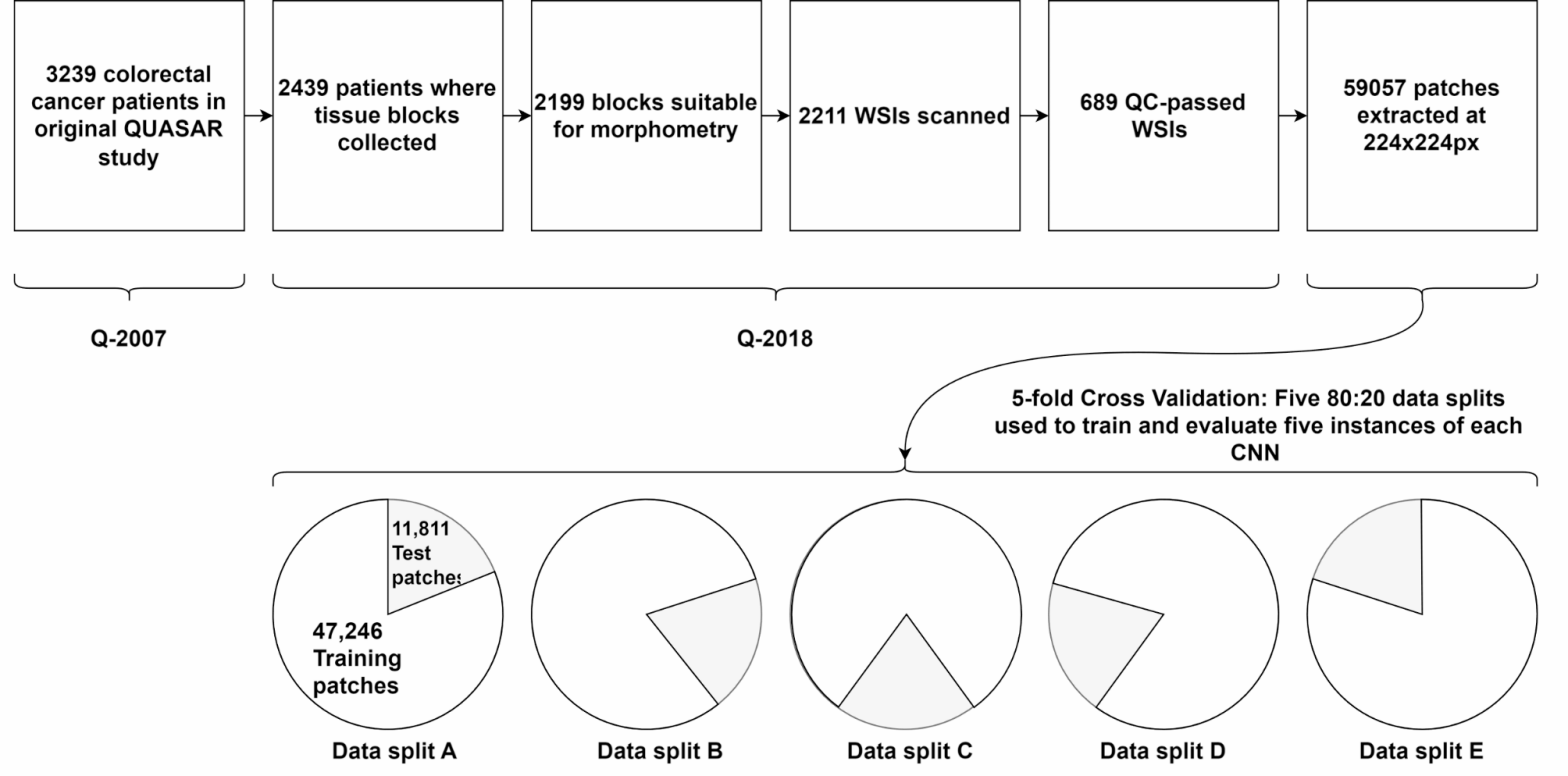
Provenance and allocation of data used in this study. Q-2007: Patients in original QUASAR trial^28^. Q-2018: Patient data used in follow-on study^4^ and filtered according to Quality Control criteria^29^.

**Fig. 2:**
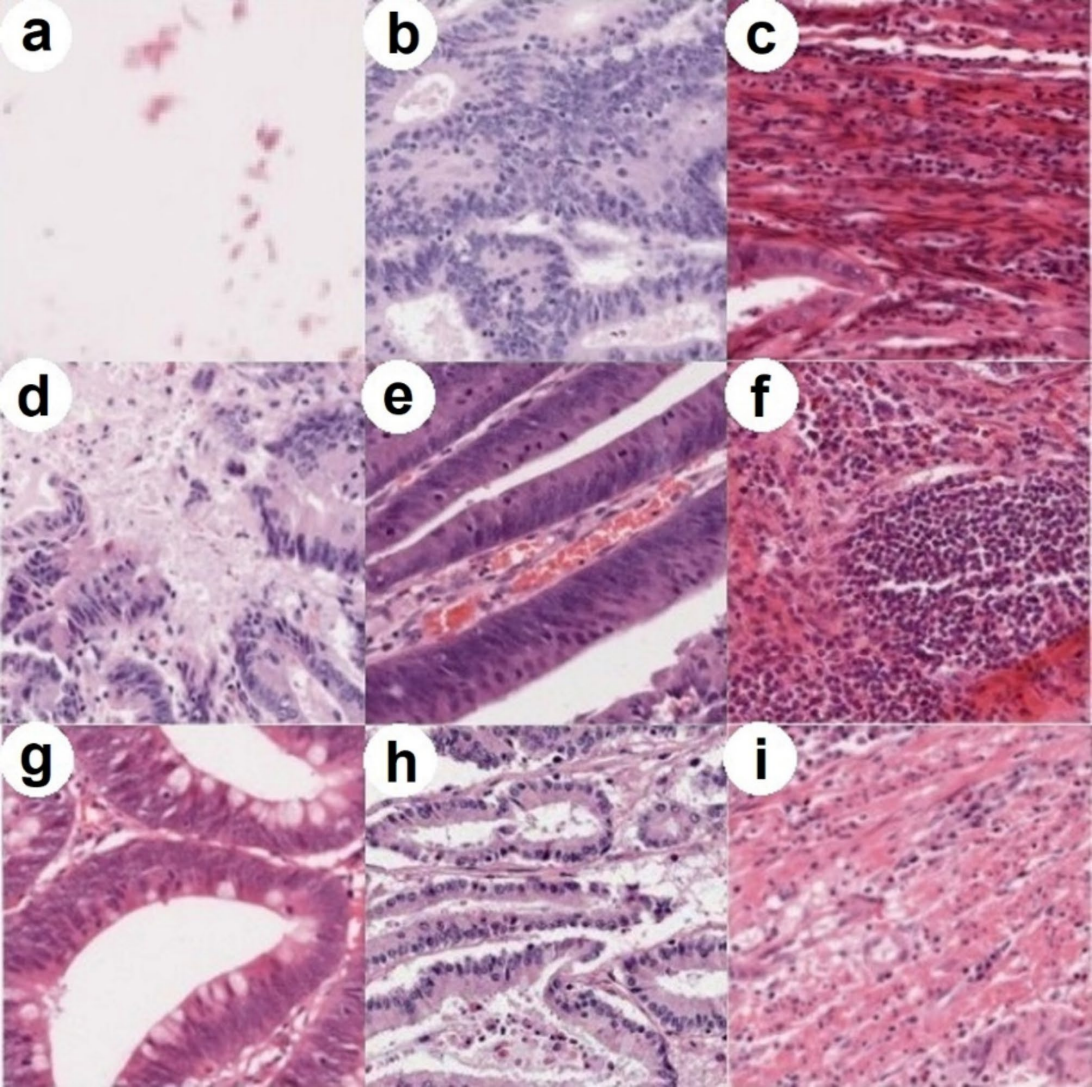
Patches extracted from WSI at ground truth sampling locations (size 448 × 448px shown). Tissue types: (**a**) Non-informative, (**b**) tumour, (**c**) stroma/fibrosis, (**d**) necrosis, (**e**) vessels, (**f**) inflammation, (**g**) lumen, (**h**) mucin and (**i**) muscle.

Figure 1 shows key stages in the sequence of data collection and selection, including filtering activities performed in the preceding studies. Example patches of each tissue class are shown in Fig. 2.

### FAL-CNN predicts tissue patch class in WSI analysis

**Fig. 3:**
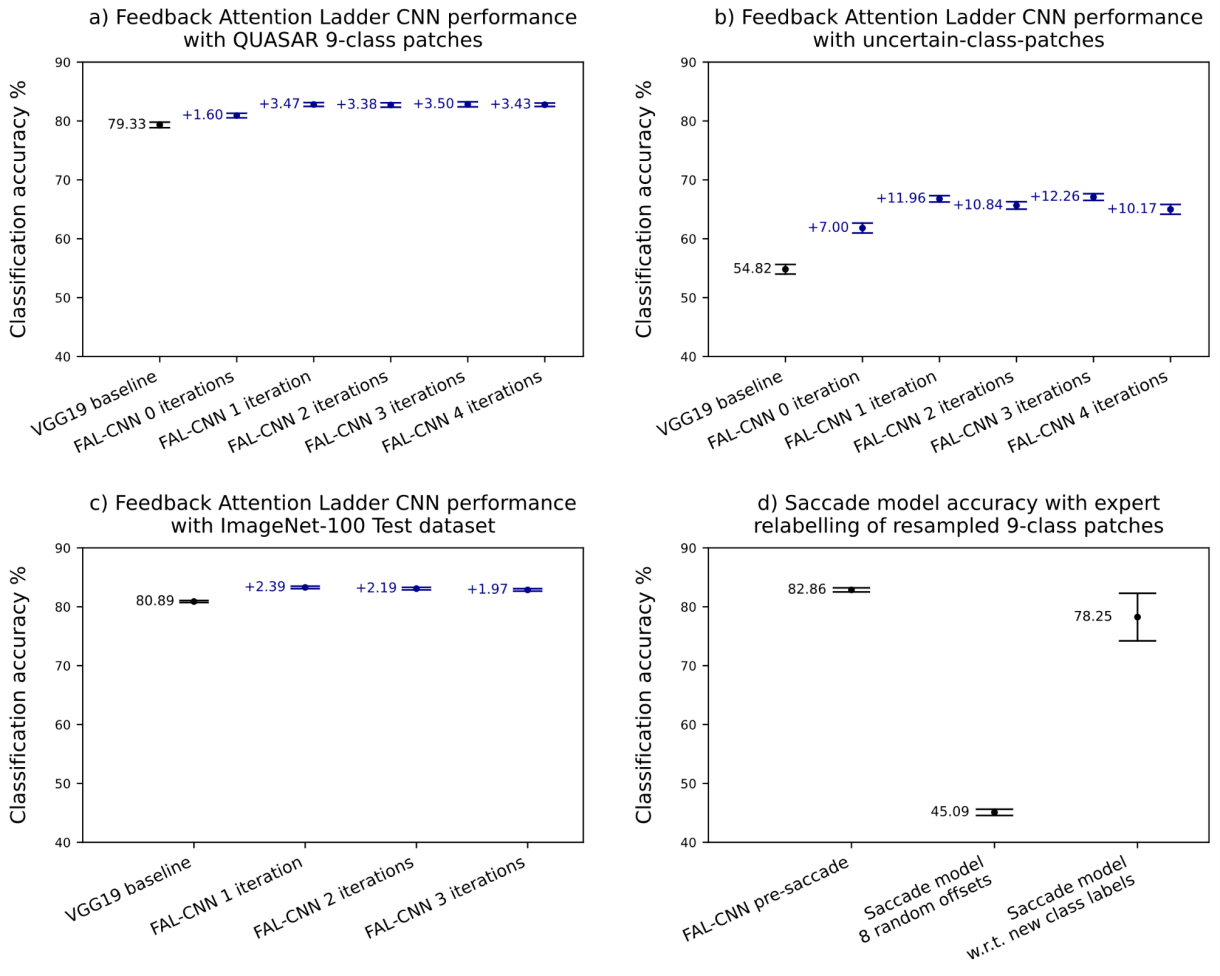
(**a**) Classification accuracies with 95% confidence intervals(CI, *N* = 30) for FAL-CNN models relative to VGG19, with 9-class colorectal cancer patches derived from QUASAR study data^4,28^. (**b**) Classification accuracies with 95% CI for FAL-CNN models with uncertain-class-patches dataset. (**c**) Classification accuracies with 95% CI for FAL-CNN model with ImageNet-100 Test dataset. (**d**) Classification accuracies with 95% CI for FAL-CNN and saccade models.

Patch-scale classification for WSI analysis commonly uses traditional feedforward CNNs such as VGG19. Our FAL-CNN (Fig. 8) adds multiple feedback pathways in a novel ladder configuration, to generate spatial masks which influence sensitivity at multiple stages in the feedforward encoder path. This approach yielded increases of approximately 3.5pp in classification accuracy with 9-class colorectal cancer patches, using 1 to 4 feedback iterations (Fig. 3a). An increase of 1.37pp was observed with zero feedback iterations, involving only the feedforward pathway with an additional fully connected (FC) neural layer.

An intentionally adversarial *uncertain-class-patches* subset was extracted from the 9-class CRC dataset, using patches for which the VGG19 reported a high probability for 2 or more output classes simultaneously. Mean classification accuracies with 95% CI were measured by invoking each model under test against 30 random subsets of a hold-out test split of *uncertain-class-patches*. Figure 3b shows an increase in accuracy of 11.96pp with the 1-iteration FAL-CNN, relative to the VGG19 baseline. The highest increase of 12.26% was seen with the 3-iteration variant.

We obtained p-values of $\:p<0.001$ for the above results, using the Wilcoxon Rank Sum Test.

### FAL-CNN predicts image class with ImageNet-100

We trained FAL-CNN and VGG19 models with the ImageNet-100^31^ training set. Models were then evaluated with the ImageNet-100 test set. The greatest increase relative to the baseline VGG19 was 2.39pp (Fig. 3c), using the 1-iteration FAL-CNN variant. We obtained p-values of $\:p<0.001$ for these results, using the Wilcoxon Rank Sum Test.

### FAL-CNN spatial attention distributions predict informative image regions

**Fig. 4:**
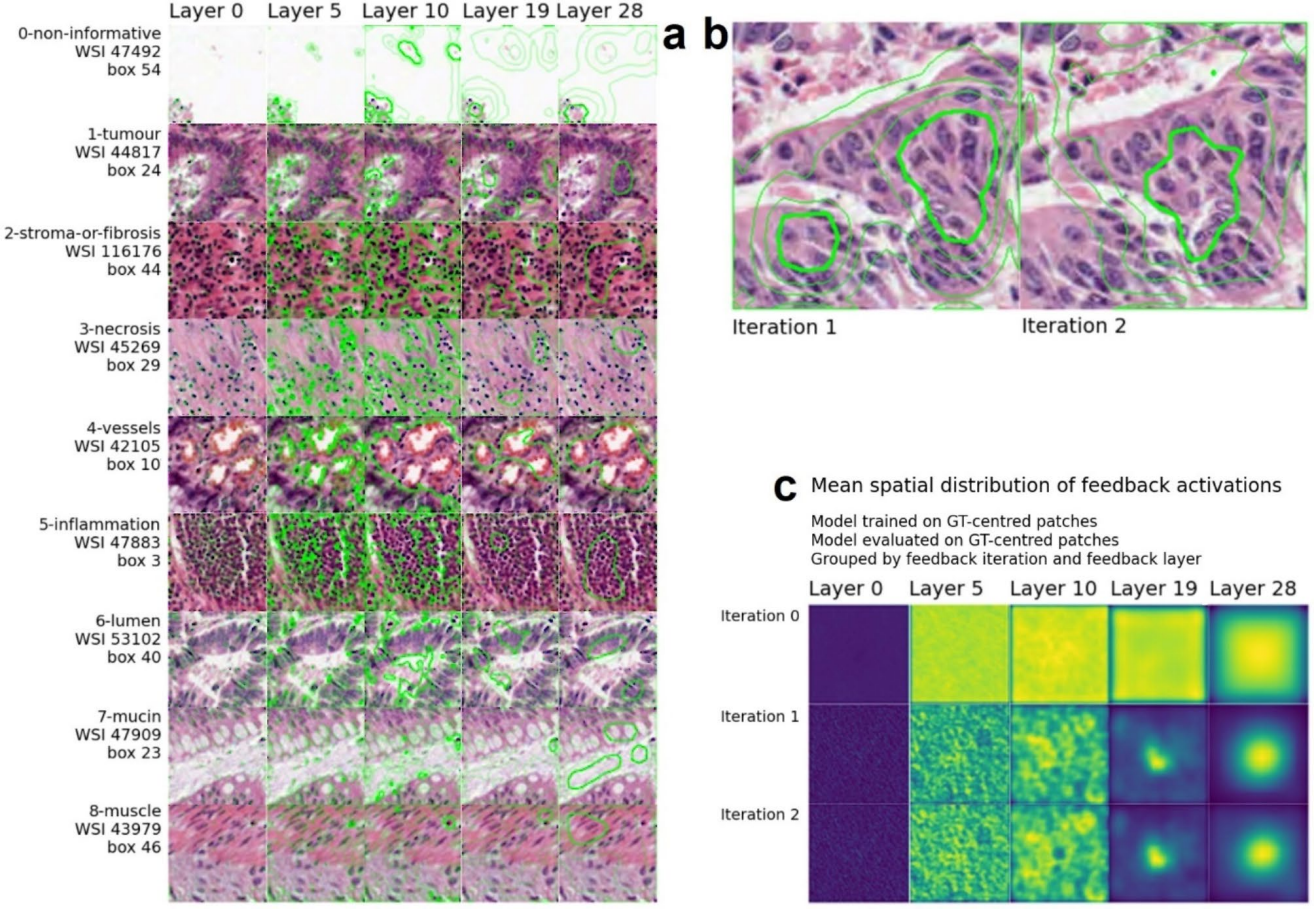
(**a**) Feedback attention contours by layer, for one sample patch of each tissue class; (**b**) Ground truth patch labelled as tumour, with contours representing mean spatial attention in FAL-CNN for feedback iterations 1 and 2, at layer 28 in encoder path; (**c**) Mean spatial feedback activations over multiple patches, grouped by layer and feedback iteration. Feedback layer numbers refer to module index in the VGG19 encoder prior to inserting feedback attention modules (FAM).

To visualise spatial correlations between input image features and attention distributions generated within the FAL-CNN model, we superimposed contour plots, representing mean spatial attention distributions at each feedback layer, upon example patches of each colorectal cancer tissue class (Fig. 4a). In lower layers, the contours follow textural image features such as cell nuclei. In higher layers, the contours overlay regions of tissue that are characteristic of the patch class.

Figure 4b shows a patch labelled as *tumour*, overlaid with attention distributions at layer 28 generated during feedback iterations 1 and 2 of a 2-iteration model variant. In both images, cells within the 80% contour have the dark, densely packed nuclei characteristic of tumour tissue.

We then combined feedback activation maps for multiple input images, to examine overall attention distributions at each feedback layer. Figure 4, C shows mean spatial distributions grouped by feedback layer and iteration. Over multiple iterations, attention in higher layers is increasingly focused on the central pixel.

**Fig. 5:**
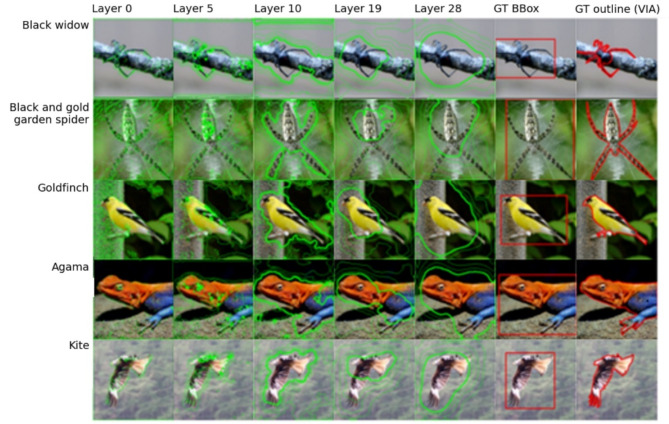
Feedback attention contours and ground-truth annotations for ImageNet-100 sample images, arranged by class and layer. Rightmost two columns show human-generated GT bounding boxes and object outlines.

Contour plots were also created for ImageNet-100 examples (Fig. 5), to test our model’s transferability to other datasets, and to examine its attentional behaviour in relation to readily identifiable image features. Feedback contours at higher layers enclosed the target’s head, body, wings or legs, and were approximately concentric with manual annotations for bounding box or object outline. Lower level feedback activations overlaid fine-grained structures such as feathers and informative background textures such as a spider’s web.

### Saccade model recentres ImageNet-100 image on informative object features

**Fig. 6:**
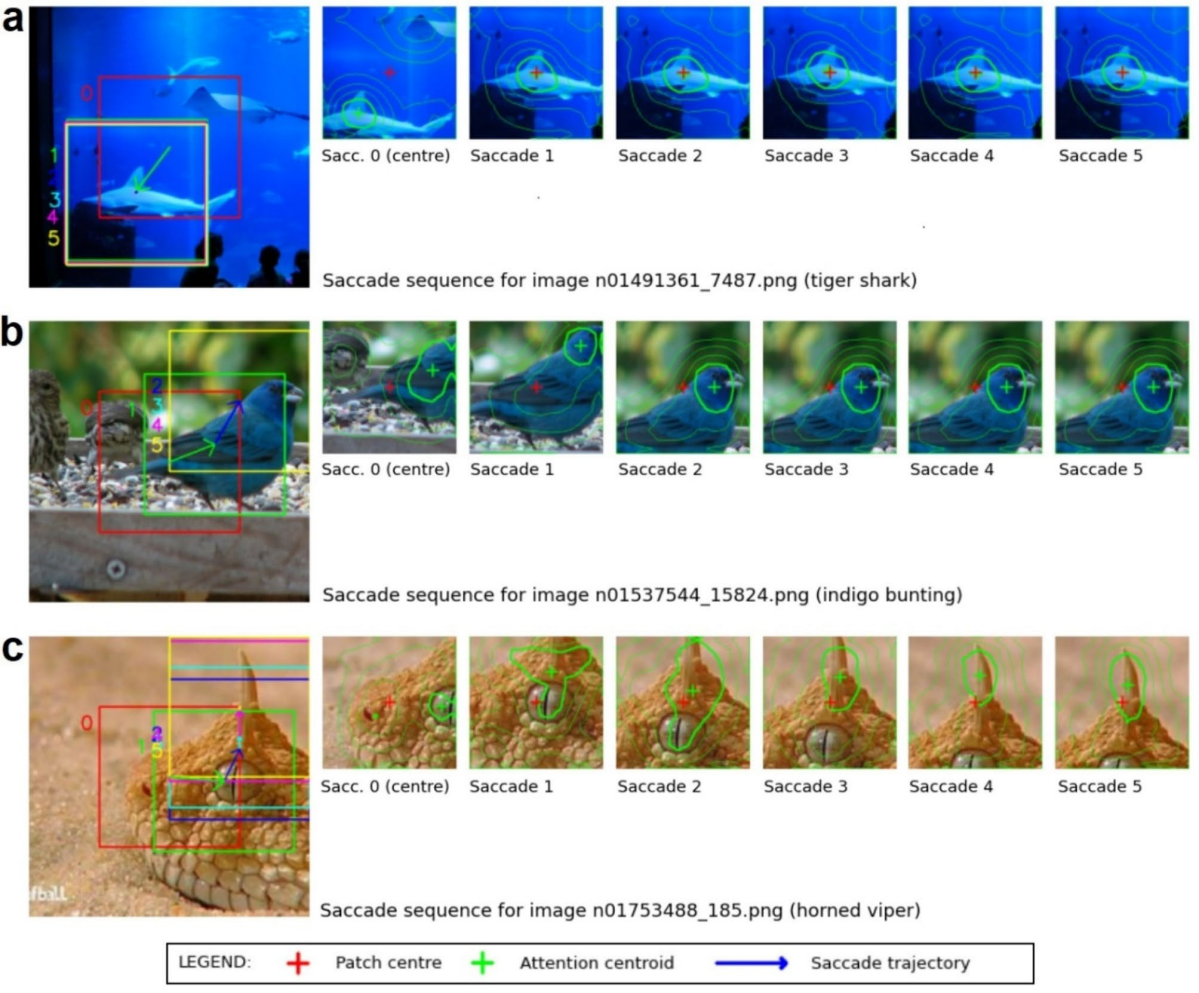
Example saccade sequences for ImageNet-100 classes (**a**) tiger shark, (**b**) indigo bunting and (**c**) horned viper.

Our saccade model iteratively samples an image patch from a larger background, using an embedded FAL-CNN to generate attention distributions which determine the next patch location. Thus the saccade process converges on informative features of the target object. Figure 6 illustrates this behaviour with examples from ImageNet-100. Here, the saccade model attends most strongly to distinguishing features such as a shark’s dorsal fin (Fig. 6a) or the horn of a horned viper (Fig. 6c). In Fig. 6b the model has successfully located the bird’s head, despite this being initially outside the sampling region.

### Saccade model recentres patch image on confirmed tumour tissue

**Fig. 7:**
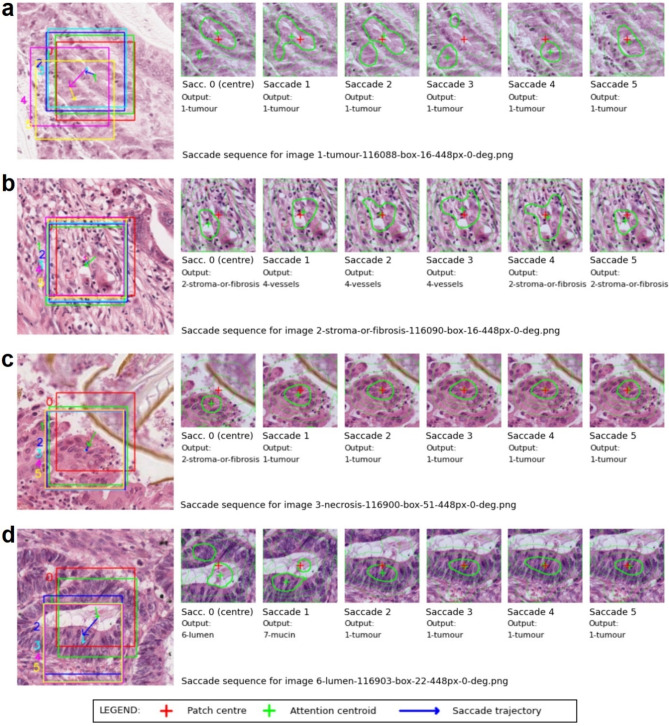
Example saccade sequences for QUASAR patch classes (**a**) tumour, (**b**) stroma, (**c**) necrosis and (**d**) lumen.

Figure 7 shows saccade sequences for examples of 9-class CRC patches, of class *tumour* (a), *stroma* (b), *necrosis* (c) and *lumen* (d). In each case, the sampling region tracks to centre the inner patch on the tissue region most strongly attended by the FAL-CNN model. The “Saccade 0” image represents the initial sampling location.

The FAL-CNN’s predicted class output is reported for each image. In Fig. 7a and b, the saccade process has converged on tissue consistent with the original class label. Contrastingly, in Fig. 7c and d, the saccade behaviour has centred the inner patch on regions of tumour nuclei, and the final predicted class has changed to *tumour*.

### Saccade model *tumour* predictions agree with expert relabelling

Figure 3, D shows the classification accuracy of the 1-iteration FAL-CNN, prior to any saccade movement, with that of a saccade model after 8 ‘random walk’ movements, and a saccade model after 8 attention-guided saccades. The post-saccade patches were labelled by a pathologist according to tissue class at the centre of the new patch, as the original class label was no longer applicable at this location. Over 400 patch images, we observed a 78.25% (95% BCI 74.21 to 82.29%) rate of agreement between the predicted class in the final saccade and the pathologist’s new class label.

For Table 1 we calculated rates of agreement between the saccade model’s predicted class and the pathologist’s label, grouped according to the new class label. 266 out of 400 images were labelled as *tumour*, with an agreement rate of 93.23% with the saccade model output (95% BCI 90.21 to 96.25%).

**Table 1:** Per-class breakdown of agreement rates between saccade model output and relabelled final sample location (9 input classes).

Expert-assigned label for post-saccade patch image	Number of patch images	Total in agreement with saccade model output class	Mean agreement rate %	Binomial probability confidence interval %
All	400	313	76.90	72.81 to 81.00
0-non-informative	28	4	14.29	1.32 to 27.25
1-tumour	266	248	93.23	90.21 to 96.25
2-stroma-or-fibrosis	43	24	55.81	40.97 to 70.66
3-necrosis	16	8	50.00	25.50 to 74.50
4-vessels	14	11	78.57	57.08 to 100.00
5-inflammation	8	3	37.50	3.95 to 71.05
6-lumen	16	8	50.00	25.50 to 74.50
7-mucin	9	7	77.78	50.62 to 100.00
8-muscle	0	0	0.00	-

## Discussion

**Fig. 8:**
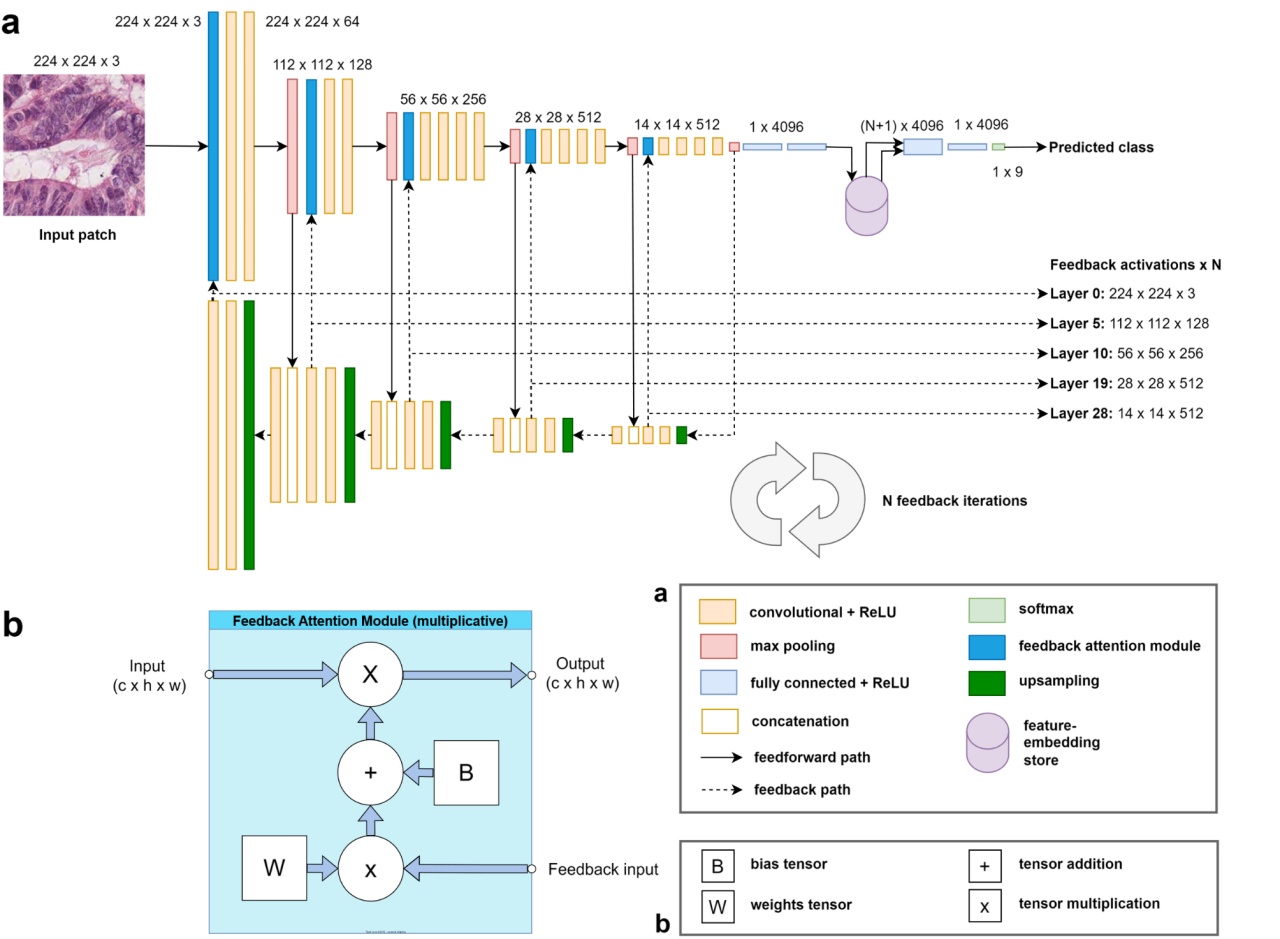
(**a**) Feedback Attention Ladder CNN (FAL-CNN) classifier, with additional feedback activation outputs supporting attention visualisation; (**b**) Multiplicative Feedback Attention Module (FAM) used to apply multi-channel spatial feedback activations to feedforward path.

We have developed a novel, biologically inspired neural network for pathology patch classification: the Feedback Attention Ladder CNN, or FAL-CNN (Fig. 8). We used a folded U-Net^24^-derived structure, whose decoder generates feedback activations which control spatial attention at multiple convolutional levels in the encoder. Our model uniquely combines top-to-bottom feedback with local feedback paths encompassing convolutional groups at each spatial scale-level. Feature embeddings were aggregated over multiple iterations of feedforward and feedback processing, using a Feature Embedding Store (FES), inserted between fully connected (FC) layers, to inform the model’s output class prediction.

The FAL-CNN configuration yielded significant increases in classification accuracy with multiple datasets, relative to the feedforward-only VGG19 architecture which supplied the encoder backbone in our model. This claim is supported by non-overlapping 95% confidence intervals and p-values by Wilcoxon Rank Sum Test of $\:p<0.001$. When trained with ImageNet-100, our model was 2.39pp more accurate than the VGG19 (Fig. 3c). With 9-class CRC pathology patches, we saw a 3.50pp increase (Fig. 3a).

We further assessed the FAL-CNN with the *uncertain-class-patches* subset of CRC patches, to examine model behaviour with adversarial data with which the VGG19 had reported high probabilities for two or more tissue classes. With this data, use of the feedback architecture increased classification accuracy relative to VGG19 by 11.96pp with 1 feedback iteration, and 12.26pp with 3 iterations (Fig. 3b). Even with no feedback, an improvement of 7.00pp was observed, suggesting that the additional hidden FC layer at the FES output adds extra capability in discriminating the object classes implicit in the feature embeddings at this level in the model, regardless of whether feedback is applied. The addition of feedback to this enhanced feedforward model results in further significant gains in accuracy. This concurs with studies^20–22^ which found that top-down attention improves discrimination in CNNs, especially for images with heterogeneous or ambiguous content.

For all datasets, a single feedback iteration delivered a significant increase in accuracy relative to the feedforward-only backbone. Further iterations sustained this accuracy level, suggesting that the hybrid feedback system’s “ladder” of multiple cross-connections between the feedforward and feedback paths acts to stabilise the feedback activations over multiple iterations. Our novel FES stage ensures that the output class prediction uses an optimum combination of feature embeddings from each iteration.

Spatial distributions of attention activations at each feedback level in the FAL-CNN showed visual correlation between feedback attention maps and salient image features. With ImageNet-100 (Fig. 5), the higher feedback layers highlighted larger features of the target object, such as a bird’s head or a shark’s dorsal fin. In lower layers, attention distributions followed finer details such as feathers, scales and informative background textures.

Similarly, when using 9-class CRC pathology patches, feedback activations showed that our model attended to informative tissue features at multiple scale levels (Fig. 4a). In lower layers, the feedback contours were aligned with nuclei and other textural features. In higher layers, structures and regions of tissue associated with classes such as *tumour* and *stroma* were highlighted. In this way our model contributes to Explainable AI (XAI), by highlighting human-recognisable structures that contribute to its class prediction.

When averaged over multiple patches, the attention distributions revealed a central focus (Fig. 4c), consistent with the annotating pathologist’s behaviour in applying a class label to a single nominal pixel whilst examining nearby tissue structures for context (the initial annotation instructions were to assign a label corresponding to the central pixel in the image, marked with a crosshair).

Our saccade model exploited this tendency by resampling the input patch to align the most strongly attended image features with the centre region where the model is most sensitive (Fig. 6). This behaviour is analogous to foveal vision in humans, and enabled our model to converge on informative structures such as head, eyes or fins in ImageNet data or tissue structures relevant to tumour tissue in CRC patches. Notably, this occurs even when the feature is initially outside the crop region, such as in Fig. 6b where the saccade mechanism locates the initially cropped head of an indigo bunting. This process is not reliant on the storage of previous points of interest, but follows an ascending attention gradient towards the most salient features.

The classification accuracy of the embedded FAL-CNN after the final saccade was significantly higher (supported by 95% CIs) than was achieved with random movements, confirming that the FAL-CNN’s attention regions represent salient features of the input patch, supporting their usefulness for XAI applications.

With 9-class colorectal cancer images, the saccade model frequently recentred the patch sampling region on neighbouring regions of tumour. Expert relabelling was carried out on 400 post-saccade patches, to identify the tissue type corresponding to the new patch location. The model’s final class prediction agreed with the new class label in 76.9% of cases (Fig. 3d). For *tumour*, representing 62% of the expert labels, the agreement rate was 93.23% (Table 1). Compared to typical inter-pathologist agreement rates of approximately 85% when manually labelling CRC patches^30^, this represents accurate identification of tumour tissue.

Lower agreement rates were observed for other tissue classes, many of which were sparsely represented in the relabelled dataset (Table 1). We acknowledge this as a limitation of this study and recommend further analysis using a larger dataset, rebalanced to ensure all tissue classes are strongly represented in the expert-relabelled model output. Nonetheless, we have demonstrated a perceptual model that reliably tracks to nearby tumour tissue in a WSI, with potential application as an XAI-supported diagnostic tool which yields candidate positions for a pathologist to examine.

We acknowledge that saccade model execution involves the extra computation of multiple executions of the nested FAL-CNN model, per patch. However, given its ability to track to nearby tumour in a larger ‘parent’ tile, we expect that the per-patch execution time will be mitigated by the reduced number of WSI tiles being examined to obtain a WSI-level result.

FAL-CNN used the established VGG19^8^ classifier as its feedforward encoder, chosen for its linear structure and its performance in WSI analysis^9^. We have shown that the addition of our novel multi-level feedback ladder, with FES, significantly and substantially boosts the model’s accuracy. We anticipate that this approach will also beneficially augment recent feedforward CNN models in the ImageNet challenge^31^ such as EfficientNet^8,32^, in combination with novel optimisation approaches^33,34^.

The FAL-CNN has potential application in DP workflows involving WSI processing, wherever a feedforward CNN is currently specified for tile classification or feature extraction. Examples include TSR evaluation^9^, where multiple tiles are classified to assess proportions of tissue types, and MIL applications that use patch-level feature embeddings with weakly supervised methods for WSI-level categorisation^12,13,35,36^. It is expected that our feedback-enhanced model would enhance the accuracy of such systems.

In conclusion, our biologically inspired FAL-CNN feedback attention method improves CNN performance with cancer pathology images. Our saccade model enables us to validate the FAL-CNN attention outputs for XAI purposes, and seeks out tumour regions in pathology images.

## Methods

### Ethical approval

All methods were carried out in accordance with relevant guidelines and regulations and all experimental protocols were approved by a named institutional and/or licensing committee. This current work is covered under NHS ethical approval under Leeds West REC 05/Q1205/220 for analysis of digital pathology images, granted by NHS Health Research Authority, Yorkshire and the Humber Leeds West, previously known as Leeds West Research Ethics Committee. Patients gave informed written consent for their participation. All methods were carried out in accordance with relevant research guidelines at Leeds Teaching Hospitals and the University of Leeds, and reviewed against the Checklist for Artificial Intelligence in Medical Imaging (CLAIM)^37^.

### Participants

The FAL-CNN model was trained and evaluated in a retrospective study using 9-class patch images extracted from WSIs of colorectal cancer sections, previously obtained during the QUASAR trial of adjuvant chemotherapy in resection surgery^28^. Participants, numbering 3,239 with median age 63 (IQR 56–68) years from 19 countries, from May 1994 to December 2003, had undergone resections of colon or rectal cancer and were randomly assigned to receive additional chemotherapy. Written consent was obtained from participants before randomisation. The full selection process is detailed in the QUASAR article^28^. A later study^4^ used a sub-group of 2439 patients from UK centres, from whom tissue blocks were available for analysis, yielding 2211 WSIs which were re-used in this current work.

### Data extraction

#### QUASAR 9-class patch images

Haematoxylin and Eosin (H&E) stained tissue was scanned at 0.49$\:\mu\:m$ per pixel using a Lecia Biosystems Aperio XT scanner system with JPEG 2000 compression at 49.09 compression ratio and a quality factor of 30^4^. A set of 689 WSIs that satisfied quality control criteria^29^ for slide mounting and scanning quality were used in our work.

A trained biomedical scientist under the supervision of a pathologist^4^ had classified tissue at approximately 50 points per WSI, using a triangular grid assigned using a RandomSpot^5^ algorithm within a 3 × 3 mm ‘virtual biopsy’ region representing maximum tumour density near the interior bowel wall. Nine tissue classes were represented: *non-informative*,* tumour*,* stroma or fibrosis*,* necrosis*,* vessels*,* inflammation*,* lumen*,* mucin* and *muscle.* For our previous work^9^, we extracted 224 × 224px patches centred on the pathologists’ sampling locations, yielding 59,057 images for training and evaluation. These were reused here to train the FAL-CNN.

#### QUASAR 9-class “uncertain class” patch images

Feedback attention has been shown to assist in distinguishing subjects in cluttered, heterogeneous images^20–22^. To assess the FAL-CNN against similarly challenging data, we extracted patches from the 9-class dataset for cases where a trained feedforward VGG19 model returned high probabilities at two or more class outputs. Patches were selected where the highest class probability $\:{P}_{A}$ and the second highest class probability $\:{P}_{B}$ satisfied the condition in Eq. 1:

1 $$\:\begin{array}{c}\frac{{P}_{A}-{P}_{B}}{{P}_{A}}<0.25\end{array}$$

The VGG19 was trained for this purpose using the training set of a specified train/test data split. Later assessment of feedback models against the resulting *uncertain-class-patches* dataset was performed using models trained with the same training set, before the models were evaluated with the complementary test set. This ensured that evaluation used patch images that were unseen during model training and data extraction, to mitigate against overfitting.

#### ImageNet-100

To evaluate our model’s generalisability to diverse data, images of 100 classes of birds and animals were downloaded from ImageNet-100^38^, a subset of the popular ImageNet-1k data set^31^. A total of 130,000 files were allocated for model training, with a further hold-out set of 5,000 for evaluation.

### Feedback attention ladder CNN

Our proposed Feedback Attention Ladder CNN (FAL-CNN) uses an architecture analogous to a folded U-Net, where the decoder outputs supply feedback activations in the form of spatial attention masks that fold back into the feedforward encoder path (Fig. 8a). The encoder is based on the VGG19 classifier model. Feedback activations are applied before the first convolutional layer in each scale-group, using a multiplicative Feedback Attention Module (FAM; Fig. 8b).

The forward skip connections, associated with the U-Net architecture, alternate with the feedback connections in a ladder-like structure. The multiple ‘rungs’ facilitate feedback within local convolutional groups, as used by CORnet^21,22^. Simultaneously, the feedback decoder path provides a top-to-bottom feedback path, allowing attention masks in lower encoder layers to be derived from high-level activations near the encoder output. Our model supports multiple iterations of this feedforward and feedback processing.

In a further enhancement, we aggregate the feature embeddings generated by each feedforward pass in a feature embedding store (FES). This captures encoder outputs from the initial forward pass, and after each subsequent feedback iteration. The FES was implemented as a tensor with dimension $\:BC(N+1)$, where $\:N$ is the number of feedback iterations, $\:C$ is the number of channels in the fully connected (FC) layers of the model, and $\:B$ is the image batch size. An additional FC layer was inserted after the FES to reduce the stacked embeddings to size $\:BC$. A final 9-channel FC layer and *softmax* module, as used in VGG19, then generate the output class prediction.

Model configurations with 0 to 4 feedback iterations were trained with the QUASAR-derived 9-class patches. Weights in the encoder path were initialised from corresponding layers in an ImageNet-pretrained VGG19 downloaded from the Pytorch ‘Model Zoo’^39^. Decoder weights were randomly initialised.

Models were trained using Stochastic Gradient Descent (SGD) with Cross Entropy Loss for 200 epochs, with an initial learning rate (LR) of 0.0003 and momentum of 0.9. LR scheduling, reducing the LR by a factor of 0.7 every 30 epochs, was found to give optimum loss convergence.

### Statistics

Five-fold cross validation (CV) was used with each model configuration. QUASAR-derived patches were grouped by originating WSI. Five data splits were defined, with an 80%:20% split between training and test sets, such that each test set contained patches derived from mutually exclusive collections of WSIs.

Mean classification accuracies were measured against the five test sets, in each case using a model version that was trained using the corresponding training set. Bootstrapping was performed by splitting each test set into six sub-groups and performing inference on each patch in the sub-group. Thirty mean accuracy points were thus generated, supporting calculation of overall mean accuracy with 95% confidence intervals.

Error bars in our results represent 95% confidence intervals. These were compared between baseline VGG19 and FAL-CNN configurations. This is a one-tailed test, with the expectation that the FAL-CNN results distribution has a higher mean than those for the VGG19 baseline.

We calculated p-values using the Wilcoxon Rank Sum test, with the SciPy Python library function *scipy.stats.ranksums()*.

### Visualisation of spatial attention distributions

Spatial distributions of attention activations were expressed as contour plots, superimposed on the input patch image. For each feedback layer in the FAL-CNN model, per-channel spatial activations were combined into a mean $\:{H}_{l}\times\:{W}_{l}$ array, where $\:{H}_{l}$ and $\:{W}_{l}$ are the spatial dimensions of the encoder at level $\:l$. The array was then normalised to the range $\:\left[\text{0,1}\right]$ and scaled with interpolation to generate a smooth heatmap-like distribution at the $\:224\times\:224px$ input scale. Contour polygons were derived from the heatmap distributions and combined with the patch image using OpenCV *findContours* and *drawContours* functions^40^. Separate plots were created for each iteration and feedback layer in a model using 2 feedback iterations.

A consultant pathologist subsequently performed a qualitative analysis of contour plots for randomly selected patches of each class, for an informed visual assessment of the correlation between attention ‘hotspots’ and informative tissue structures. Contours generated for layer 28 were preferentially examined, as these were found to enclose larger regions of cells, allowing structural context as well as cell types to be assessed.

To analyse attention distributions across multiple patch images, heatmap plots were generated for each model layer by combining mean activations from multiple executions of the FAL-CNN model. These were grouped by feedback layer and feedback iteration, for a model using 3 iterations. Each $\:224\times\:224px$ output was normalised then converted to an RGB image with a blue-to-yellow *viridis*^41^ colour mapping, which is perceptually uniform and offers good visual contrast.

Contour plots were also generated using a FAL-CNN trained and evaluated with ImageNet-100, to facilitate an intuitive, qualitative assessment of the model’s attention regions in relation to image features. Ground truth bounding box annotations were downloaded from the ImageNet challenge site^31^ and plotted for comparison. Object outlines were annotated online using the VGG Image Annotator^42^ (VIA) for one random sample of each ImagNet-100 class.

### Saccade model

**Fig. 9:**
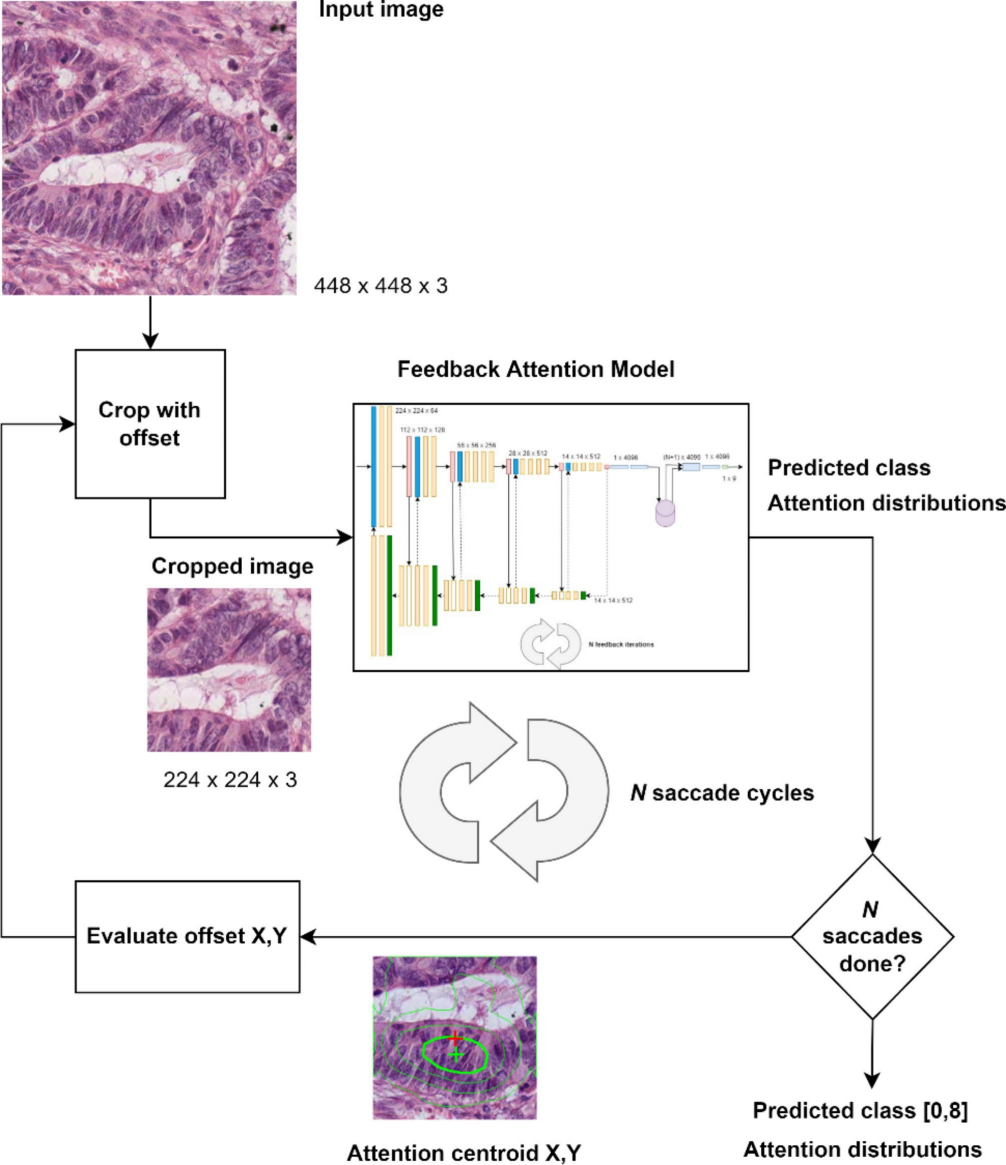
Saccade model with tumour patch.

This further model (Fig. 9) was developed to explore the effect on FAL-CNN performance of resampling the input patch to align informative tissue regions, as highlighted by spatial attention distributions from the FAL-CNN, with the patch centre. We expected that this behaviour, emulating saccades in animal vision, would cause the model to track towards tissue of interest in pathology patches, and towards identifying features of objects in ImageNet samples.

An input size of $\:448\times\:448px$ was used, from which the central $\:224\times\:224px$ region was initially sampled. This was applied to a one-iteration FAL-CNN, to generate an initial class prediction and associated feedback attention activations. A centre of attention (CoA) was derived from the highest attention layer, using the centroid of the 80% attention contour as a proxy for peak attention. A new $\:224\times\:224px$ region, centred on the CoA or random offset, was then sampled from the input image. This image was used as the FAL-CNN input for the next iteration of the Saccade model. Up to 10 such iterations were performed. Model outputs included vectors of predicted classes and cropped input patches for each cycle.

To compare attention-guided saccade model performance with that of a ‘random walk’ approach, we developed a further model variant that applied random horizontal and vertical offsets in a range of $\:\pm\:112px$ per saccade cycle.

The model behaviour is summarised by the following algorithm:

- Input:
- 448 × 448px image.
- Sample central 224 × 224px patch from input image.
- For each of N saccades:
  - Apply sampled patch to feedback attention model.
  - Derive centre point of mean feedback activation.
  - Calculate offset from centre of patch.
  - Sample new 224 × 224px patch from input image with this offset.
- Return:
- Arrays of predicted class and feedback activations per saccade.

The saccade model was executed for 9-class CRC patches using 8 saccade cycles. Images were randomly sampled from the hold-out set associated with the original train/test split used in training the embedded FAL-CNN model, so that only unseen patches were used in evaluation. Classification accuracy was measured over 30 such random sample sets, to obtain mean and 95% CI values.

A consultant pathologist reviewed patch images at the final sampling locations of the 8-saccade model for 400 randomly selected 9-class CRC patches. The model’s class predictions were recorded for each patch after the 8th saccade. Each new patch was labelled according to tissue surrounding its centre pixel. Rates of agreement were recorded per class and across all patches.

The saccade model was also evaluated with images from the ImageNet-100 hold-out set, square cropped and scaled to $\:448\times\:448px$, using a one-iteration, ImageNet-100 trained FAL. Thirty random sample sets were used to calculate mean classification accuracy with 95% confidence intervals (CI).

For selected patch and ImageNet input images, image sequences were plotted showing the regions sampled in each saccade cycle in a 5-saccade model, with the corresponding CoA location and output class prediction.

## Data Availability

Pathological image data was obtained by request from the authors of the original research papers^4^. ImageNet100 data that support the findings of this study are available from Kaggle at https://www.kaggle.com/datasets/ambityga/imagenet100.
